# Perceptions regarding making household air pollution a routine topic for health education during antenatal care: A qualitative study in Eastern Uganda

**DOI:** 10.1371/journal.pgph.0003973

**Published:** 2025-09-15

**Authors:** Joshua Epuitai, Katherine E. Woolley, Rose Chalo Nabirye, Julius N. Wandabwa, Joseph Odula, Ivan Lyagoba, G. Neil Thomas

**Affiliations:** 1 Department of Nursing, Faculty of Health Sciences, Busitema University, Mbale, Uganda; 2 Institute of Applied Health Research, University of Birmingham, Birmingham, United Kingdom; 3 Department of Obstetrics and Gynecology, Faculty of Health Sciences, Busitema University, Mbale, Uganda; PLOS: Public Library of Science, UNITED STATES OF AMERICA

## Abstract

In Uganda, pregnant women do not routinely receive health education during antenatal care regarding exposure to household air pollution (HAP). The study was conducted to explore perceptions regarding what would be needed to incorporate HAP as a routine topic for health education during antenatal care. The study was based on the capability, motivation, opportunity-behavior (COM-B) model. Qualitative interviews were conducted among healthcare workers and pregnant women attending antenatal care at Mbale Regional Referral Hospital in Uganda. Thematic analysis was used to identify key themes. Capability to provide health education on HAP emanated from health system factors (e.g., lack of capacity, workload, time constraints) and behavioral factors (e.g., HAP not seen as a major risk factor for adverse pregnancy outcomes). Capability to adopt cleaner fuels following health education was thought to be affected by willingness to adopt short-term interventions ahead of cleaner fuels alternatives, cost/affordability constraints, unwillingness to change, and socio-cultural concerns. Socio-economic constraints, weather and safety concerns were thought to affect women’s capability to open doors/windows and cook outdoors following health education. Participants were motivated to provide/receive antenatal health education because of their need to reduce adverse birth outcomes caused by HAP, acceptability of HAP as a topic for education, and the perception of healthcare workers as role models. Training midwives about HAP, using innovative teaching aids, including prompts on HAP in the antenatal card to remind healthcare workers to talk about HAP, giving incentives to healthcare workers and involving them when designing health education about HAP were suggested to enable integration of HAP as routine topic. Our study highlights an opportunity to empower and create demand among pregnant people to adopt behaviors that could reduce exposure to HAP during ANC. Integration of HAP into antenatal care could help transition households from precontemplation and contemplation stage in the uptake of cleaner fuels.

## Background

Globally, 36% of households rely on solid biomass fuels for cooking and or lighting [1]. In Uganda, 97% of households rely on solid biomass fuels most predominately wood and charcoal for cooking and lighting [2]. Biomass fuels produce high levels of pollutants which exceed the World Health Organisation (WHO) air quality guidelines [3]. The resulting household air pollution (HAP) is associated with poor health outcomes across the life span [4]. HAP is associated with poor pregnancy outcomes including hypertensive disorders during pregnancy, stillbirth, low birth weight, and stunting [4–6]. Women also experience gender related health risks from exposure to HAP including animal bites, gender-based violence, reduced time to engage in income generating activities, and lack of empowerment [3]. These gender-related risks occur from household cooking activities and fetching of wood in wild forests [3].

Promoting adoption of cleaner fuels such as liquefied petroleum gas (LPG), are often seen as longer-term interventions, which have the potential to reduce HAP [3]. However, adoption of cleaner stoves has stalled in low- and middle-income countries (LMIC) because of concerns of affordability, accessibility, and perceived health risks of cleaner fuels [7]. Adoption of cleaner fuels is further affected by socio-cultural concerns for food taste and attachment to the three-stone stoves [7]. In LMIC, promoting behavioral change interventions such as improving ventilation (e.g., opening windows/doors), use of dry wood, and cooking outdoors termed herein as shorter-term interventions maybe more practical and feasible, as an interim measure, to make a difference now rather than waiting for the barriers of longer-term use interventions to be broken down [8].

Behavioral change interventions can reduce exposure to HAP, while they can also supplement measures to promote adoption of cleaner fuels [9,10]. This could be through creating demand for cleaner fuels and addressing the existing limited knowledge regarding the effects of HAP [11,12]. Behavioral change strategies such as health education through use of credible figures (for example, midwives) to model and promote adoption of cleaner fuels have been used to help populations adopt cleaner fuel alternatives [11]. A review indicated how behavioral change strategies reduced HAP by 31–94% in the field settings though the reduction in HAP was still above the WHO air quality guidelines [10]. Health education during antenatal care could be effective in changing behaviors (e.g., outdoor cooking, opening windows or doors while cooking), shaping knowledge and risk perception about the effects of HAP, and in motivating adoption of cleaner fuels [10,11].

In Uganda, almost all (97%) pregnant women attend antenatal care [13]. Antenatal care, therefore, is an entry point into the healthcare system where pregnant women and their families may interface, for the first time, with the healthcare system [14]. Pregnancy in itself embodies a *“teachable*” period in which pregnant women and their families maybe motivated to adopt healthy behaviors such as using cleaner fuels [15]. Utilizing antenatal care to health educate pregnant women regarding the effects of HAP may, therefore, represent a golden opportunity to reduce the impact of exposure to HAP [15]. The resulting change in behavior during pregnancy may even continue to postnatal period and beyond resulting in sustained use of cleaner fuel alternatives [15].

During antenatal care, pregnant women receive a wide range of services including education and counselling sessions [16]. Health education and promotion during antenatal care empowers pregnant women to prevent exposure to important risk factors [16]. In Uganda, clinical guidelines emphasise the critical importance of informing women regarding the dangers of smoking [17,18]. While these guidelines do not clarify on the type of smoke, HAP is a significant risk factor [3,17,18]. Therefore, incorporating HAP as a topic area for education is important in creating awareness on HAP [3]. However, currently routine antenatal health education does not include HAP [19,20]. The lack of health education on HAP extends to other pregnancy-related areas where most women do not receive critical information education during antenatal care [16,20]. The gaps in the provision of health education are attributable to lack of standards to streamline and guide the content of health education during ANC [16].

Limited studies have explored the views of pregnant women and midwives regarding what would be required to include HAP in health education during ANC. The few studies in Uganda were limited to evaluating the implementation outcomes of health education strategy on HAP and did not address de novo the issues of making HAP a routine topic for antenatal health education in a high volume health setting [21]. Therefore, this study was conducted to explore perceptions of healthcare workers and pregnant women regarding making HAP a routine topic for health education during ANC in Mbale Regional Referral Hospital (MRRH).

## Methods and materials

### Study design and site

This was a qualitative study that used descriptive approach [22]. The study was guided by the philosophical underpinnings of the capability, motivation, opportunity-behavior (COM-B) model [23]. The COM-B posits that for an individual to perform a particular behavior (e.g., conduct health education on HAP), they must have the capability, motivation and opportunity to perform the behavior [23]. Capability entails the physical and psychological ability to perform the specified behavior [23]. The capability in this study reflected the capacity to provide health education during ANC, but also the capacity to comply with advice to reduce exposure to HAP. Motivation referred to mental psychic processes that fueled the performance of a particular activity [23]. This involved automatic or subconscious motivation and reflective or conscious motivation [23]. Opportunity in the COM-B model represents the external factors that provided incentives or barriers to the performance of a particular behavior [23]. Previous studies have equally used the COM-B model to explain the uptake or the barriers to uptake of measures to reduce exposure to HAP [24].

The study was conducted in MRRH, a facility that serves the Bukedi, Teso, and Sebei sub-regions from rural and urban settings. The hospital offers comprehensive emergency and obstetric services across the continuum of maternal and child health services including antenatal care services. The antenatal care services run from Monday to Friday every week, receiving about 500 pregnant women per month. The midwives in collaboration with the attending medical personnel including the obstetrician and gynecologist are the main healthcare providers of antenatal care services in the MRRH. Antenatal health education is part of the routine services offered in the antenatal care clinic in the hospital. Midwives are primarily involved in providing health education to pregnant women. The topics for health education cover a range of areas including prevention of malaria, anemia, HIV, nutrition and birth preparedness.

### Study population and sampling strategy

The study comprised pregnant women who were attending antenatal care, midwives who were involved in provision of health education in the antenatal clinic of MRRH, nurse/midwife managers in the hospital and the medical personnel in the hospital.

Purposive sampling was used to select the participants to allow for collection of rich and in-depth data [25]. We selected healthcare workers with variable years of experience in the antenatal clinic, level of qualification or training, nature of work (involved in health education) and leadership roles in the hospital. Pregnant women, attending antenatal care, were selected based on their willingness to participate in the study. The pregnant women, who attended antenatal care, were from diverse settings which were served by the hospital. Being a qualitative study, the sample size was based on the principle of data saturation [25], which was arrived when no new information from the interviews were collected. We reached data saturation after two focused group discussions (FGDs) with pregnant women, two FGDs with nurse-midwives, and three key informant interviews (KII) with a medical officer, a midwife, and nurse/midwife manager.

### Data collection and procedure

Data was collected from 01^st^ May 2023–31^st^ July 2023. JE, JO and IL conducted the FGDs and KIIs. FGD with healthcare workers and KIIs were conducted in English, while FGDs with women were conducted in the local languages which were later translated to English. JE, JO and IL were all males and healthcare professionals with experience in qualitative interviews. An interview-guide with open-ended questions were used to guide in the discussions and the KIIs. The interviewer guide had questions about perceptions of healthcare professionals and pregnant women regarding making HAP a routine topic for antenatal care health education. We also explored the pre-requisites of integrating HAP as antenatal topic area, the inherent opportunities and barriers to integrate HAP related health education during antenatal care and the motivation/willingness of pregnant women to adopt their health education messages to use cleaner fuels and or shorter-term interventions [21]. After interviewing healthcare professionals, they were trained to build their capacity to effectively conduct impactful antenatal health education on HAP, and were given visual aids adapted from elsewhere [21]. The visual aids were distributed to be used in health education talks during antenatal care. We held a FGD with pregnant women after health educational sessions to understand their experiences and perceptions with health education about HAP.

### Data analysis and quality control

The audio recording in the local language was translated to English and transcribed verbatim by the native speaker of Luganda. Thematic analysis as described by Braun and Clarke was used in the data analysis [26]. JE, JO and IL analyzed the data. The transcripts were read several times, while meaning units, codes, sub-themes and themes were identified. Disagreements in developing the codes were resolved through consensus. We initially used inductive approach to map out all the categories and codes in the data, while deductive approach was used to identify the themes that comprehensively represented all the codes in the data (S1 Table). The categories in the COM-B model were used to represent the themes in the study [23]. Rigor of the study findings were maintained through peer debriefing to discuss consistency in coding, bracketing of preconceived notions, and triangulation through use of multiple methods of data collection and personnel for data analysis [25].

### Ethical considerations and inclusivity in global research

Ethical clearance was obtained from the Busitema University, Faculty of Health Sciences Ethics and Research Committee (reference number: BUFHS-2022–39). Written informed consent was obtained from all the study participants. Pregnant women below the age of 18 years provided written informed consent as they were treated as the emancipated minors. Administrative clearance was sought from the MRRH. Additional information regarding the ethical, cultural, and scientific considerations specific to inclusivity in global research is included in the Supporting Information (S1 Text).

## Results

We identified three themes based on the COM-B Model framework (S1 Table). The themes included capability to provide and or receive health education on HAP, motivation to provide health education on HAP during ANC, and the opportunity which influences provision of health education on HAP.

### Theme 1: Capability to provide and or receive health education on HAP

Capability entailed individuals’ ability to engage in a certain behavior [23]. Capability was conceptualised in twofold: capacity to provide health education on HAP and capacity to receive health education about HAP. The capacity to provide health education on HAP was perceived to be related to level of knowledge and skills of healthcare workers, and the heavy work load. The ability to receive and implement health education given during ANC was influenced by affordability concerns, unwillingness to change, and perceptions about cleaner fuels.

#### What prevents the incorporation of HAP into routine ANC?

One of the major reasons why HAP was not integrated as a routine topic for health education during antenatal care was because it was seen not to pose a serious health risk to pregnant women (S1 Data). Topics like HIV, malaria, and obstetric emergencies were prioritised for health education as they were thought to cause a more serious health risk to the pregnant women than HAP. Some considered HAP a new topic, while a majority underestimated HAP as a serious health risk factor that could cause poor pregnancy outcomes.

*“….the health workers under look it may be thinking that it is not a high risk.”* (KII healthcare worker)

*“We tend to focus so much on the pregnancy and maybe the things that are on the [antenatal] card, are you using mosquito net, have you taken fansidar?”* (KII healthcare worker)

As a result, HAP was not considered a major topic for health education. In a few cases when pregnant women were informed about HAP, it was done incidentally when assessing for cigarette smoking in the household.

*“I would say it is not a direct topic given but it comes in as a kind of secondary information…. It comes just as an example but it doesn’t come as a major topic.”* (KII healthcare worker)

The capacity to provide health education on HAP was perceived to result from the healthcare providers’ lack of knowledge regarding HAP. Healthcare workers admitted lacking critical knowledge about what HAP is, its associated health effects, the ways to mitigate exposure to HAP, the ability to advice women about HAP during HAP. The inadequate knowledge resulted in the inability to perceive the need to provide the health education in the first place, but it also incapacitated provision of health education to pregnant women. Healthcare workers were not knowledgeable about HAP because they do not receive pre-service and in-service training on HAP like it was for cigarette smoking.

“*We were taught as midwives that a mother who is smoking[cigarettes], smoking can affect the growth of the baby. So, for us we have been thinking of the cigarette smoke [only], little did we know even this smoke [HAP] can affect”* (KII healthcare worker).

The heavy workload amidst the competing work interests such as the many topics to teach pregnant women were thought to decrease the likelihood to teach pregnant women regarding HAP.

*“There are many things that are really causing risks to babies or their mothers. So, you really want to capture. Sometimes you find may be the workload is too much and you find there is no time”* (KII healthcare worker).

#### What prevents pregnant women from adopting HAP messages to reduce exposure to HAP.

The affordability concerns, unwillingness to change, and perceptions that some of the shorter-term interventions would be impractical to adopt were thought to be the challenges women would face following ANC health education. These challenges were thought to affect pregnant women’s capability to receive the health education during ANC but it was also thought to affect their ability to adopt and implement measures to reduce exposure to HAP.

Affordability concerns was one of the identified reasons that could prevent pregnant women from adopting antenatal health education to reduce exposure to HAP. Although pregnant women were thought to be willing to adopt health education messages given during antenatal care, low socio-economic status was identified as the major challenge especially for adoption of cleaner fuels. Cleaner fuels such as LPG and electricity were perceived to be more expensive and unaffordable for most of the pregnant women.

*“…so that one can be a barrier because they cannot afford a better fuel that is safer because maybe it is not affordable. I think basically the main barriers could be the socioeconomic status.”* (FGD healthcare worker)

*“..electricity is very expensive ….so at times, situations catches us up and we have no other option.”* (FGD pregnant woman).

The second major barrier to adoption of health education messages given during antenatal care was unwillingness to change. Most of the pregnant women were perceived to be resistant to change because of the long history of using polluting cooking fuels without seeing immediate and direct effects of HAP.

*“I also do know there are some who will still be in doubt…who will say that no, what they are saying is not applicable. …there’s no way they can change…since they are not seeing a direct and immediate effect they may not change.”* (KII healthcare worker).

Perception that HAP had no effect on the baby was perceived to deter some pregnant women from adopting cleaner fuel alternatives.

*“…they may just say: me I am pregnant how will this smoke reach the baby? Some of them may not actually take in the information”* (KII healthcare worker).

Pregnant women feared to use cleaner fuels because they were seen to be more dangerous than polluting fuels. LPG fuel were noted to have a harmful smell, and hearsay stories of LPG explosion further dampened their interest to use LPG. This suggests that pregnant women would be reluctant to use cleaner fuels even after receiving a health education from the healthcare workers.

*“Gas is harmful because you may open it and delay to put the light on. That smell which is coming can cause problems.”* (FGD healthcare worker).

*“I have never used it but I hear if you maybe make any slight mistake, it will burn the house or it will burn the children….so me, I personally fear Gas[LPG]”.* (FGD pregnant woman).

Healthcare workers believed that shorter-term interventions would be feasible for pregnant women to implement after the health education. However, practical challenges were raised concerning implementing some of the shorter-term interventions. Cooking outdoors were thought to be impractical to adopt given the weather interruptions (windy or rainy conditions), insecurity concerns and unhygienic environment. Pregnant women who rented a single room without windows would find it difficult to follow advice given during antenatal care to open windows while cooking or even to cook outdoors at night when it was raining.

*“….For example, maybe when I am in a rental, the place is very small…the place[outdoors] is not safe for cooking. So, you decide to cook from inside. They can even steal your food [outdoors]. ….”* (FGD pregnant woman).

*“The challenge we get after doing the right thing, maybe I can open the window and wind is too much and it can put my fire off. That can force me to close the window. Secondly….the security is not here, the place is not secure. maybe I can close the window in order to be secure.”* (FGD pregnant woman).

Healthcare workers admitted the difficulty in changing the perception of pregnant women regarding adopting cleaner cooking fuel alternatives. The difficulty stretched from perceived incapacitation to adopt cleaner fuels because of socio-economic status to deeply entrenched beliefs regarding the effects of cooking fuels. As a result, healthcare workers thought that it would be a gradual and ongoing complex process. Consequently, midwives devised a gradual approach to use for health education. The end goal of health education would be to create awareness, encourage modification of the cooking environment, and promote adoption of shorter-term interventions such as opening windows/doors, outdoor cooking and use of curated wood. The gradual approach was thought not to cripple and overwhelm pregnant women but a feasible strategy in making a steady progress in creating sustained demand to reduce exposure to HAP. The health education was seen to empower pregnant women with information critical in making small and yet impactful changes. Health education was seen to remedy the lack of knowledge which strongly underlined the use of polluting cooking fuels.

*“Let us make it in such a way that it does not demand so much. It shouldn’t over demand that you must use gas…No. let us make it in such a way that first of all it brings knowledge to them, they know that wet firewood is bad and if it is making smoke, dodge it. Don’t use it. You a mother should either be aside or open the windows but don’t be at the smoke. It affects your lungs and your baby; it affects the growth of the baby even the brain.”* (KII healthcare worker).

*“…Knowledge is power. Once these mothers are given this knowledge, they will not change as per say I am going to change from charcoal to the fuel which they cannot afford…but at least…she is able to change in form of environment. If she has been cooking from the same room where she is sleeping, with knowledge and power they are able to put up a small shade that we cook from the other side which is a bit aerated and then we sleep this side. I think that is what may benefit them but if we say that we switch off from charcoal to fuel with this economic status, I think it will be gradual, not so fast”* (FGD healthcare worker).

*“….some mothers I think use some fuels when they do not know the effects to them yet they can actually afford the fuels that are a bit safer and some of them maybe use them in closed doors because of the ignorance they do not know….”*(FGD healthcare worker).

*“…You know Rome was not built in one day, with time as we continue talking, giving the disadvantages of cooking in the house they will pick the message and there will be a positive change, because we are not just going to say today do this and they do, no it will take us time…”* (FGD healthcare worker).

Besides creating awareness, healthcare workers thought that the health education should be tailor-made on what pregnant women could afford.

*“So, we are health educating and tell you if you cannot afford this and you are using this make sure you are in an open space or make sure you are in a well ventilated room,…tell them ad inform them of the dangers even when they cannot afford to get the better one.”* (KII healthcare worker).

Likewise, pregnant women noted that were willing to make behavioral changes which were in line with their socio-economic status. Following the health education, most of the pregnant women admitted that they will go on to implement shorter-term interventions which they thought were within their means and control.

*“I would really be willing to change but it depends. You know gas [LPG] is very costly and most of us cannot manage to get it. So, we are moving on with what we can manage.”* (FGD pregnant woman).

*“I will start using dry firewood, so that the smoke in the kitchen is reduced. I will also make sure that I use a proper stove that produces less smoke and also put it outside” (FGD,* pregnant woman*).*

### Theme 2: Motivation to provide health education regarding HAP during ANC

Motivation describes the mental psyche that fuel the performance of a particular behavior [23]. Motivation to provide health education during ANC was in the form of reflective and automatic processes [23]. The reflective motivation involved conscious acceptability of HAP as an appropriate topic for health education, and perception of healthcare workers as credible role models who could influence readily uptake of behaviors that reduce exposure to HAP. The automatic subconscious motivation reflected healthcare workers emotional motivation to provide health education to reduce the adverse birth outcomes that could be as a result of exposure to HAP.

#### HAP seen as acceptable topic for health education (reflective motivation).

Generally, the majority of participants thought that midwives would be motivated as they would find it acceptable to provide health education to pregnant women regarding the HAP. Enabling midwives to appreciate the need to teach pregnant women about the effects of HAP, giving an extra motivation, and the fact that midwives were already conducting health education to pregnant women were identified as one of the practical ways to make midwives accept HAP as a topic for health education. In addition, empowering nursing students to give health education was identified to solve the concerns of heavy workload.

*“So, how they will find it acceptable is if they also understand why we want to make it a routine topic. So, that is why I told you we need to first tell them why, the advantages, the risks these different fuels put to the mothers but if they do not understand the risks then it will be hard for them to accept it”* (KII healthcare worker).

*“I do think it is very possible…., they have appreciated it then probably there is a small motivation like a bottle of water for the beginning because the beginning is always not easy…”* (KII healthcare worker).

*“I think there is need to integrate it because we see that air pollution is a life-threatening thing to the mother and the baby…”* (FGD healthcare worker).

Empowering healthcare workers with information was critical in shaping their motivation to provide the health education as some of them were thought to harbor negative attitudes and socio-cultural issues. The negative attitudes and socio-cultural concerns were related to ubiquitous use of polluting fuels, the occurrence of adverse effects in the longer-term, and the less likelihood to attribute the adverse effects to polluting fuels.

*“I know some people will say but we grew up cooking….and nothing happened. It is like smoking you enjoy and nothing will happen for a short time…So, I believe there those people….there midwives, there healthcare workers or even doctors”* (KII healthcare worker).

Despite health education not being costly, healthcare workers noted that it will require commitment and motivation on their part to routinely conduct the health education, while others noted that it will cost them time and effort to convince the pregnant women to adopt cleaner fuel alternatives.

*“…it does not cost anything other than the usual commitment that you have been having to deliver it”* (FGD healthcare worker).

*“It is going to cost us some time to convince these ladies that really whatever they are using at home has some side effects… you say they are risky to you and your baby and then she is like is there any option to use other than what I have been using?”* (FGD healthcare worker).

Although pregnant women would be motivated to receive health education regarding HAP, midwives thought that the pregnant women would raise critical concerns related to solving the exposure to HAP especially given their inability to make some of the required changes. Encouraging use of cleaner fuels were thought to create cognitive dissonance among pregnant women who had been used to polluting fuels without any thoughts to any possible adverse health effects.

*“…they may be willing but of course be ready to answer many questions because they will tell you what do we do now? Because those are the things we use.”* (KII healthcare worker).

*“..But anyway, they will come up and ask that now what do you want me to use? Or these precautions [e.g., user cleaner fuels] may not work for me.”* (FGD healthcare worker).

Midwives noted that during the health education some of the pregnant women may come up with examples of pregnant women who experienced adverse effects of cooking fuels but continued to use them because of the challenges with adoption of cleaner fuel alternatives.

*“They will accept it [health education] but the challenge is how I am going to stop using it [polluting fuels]….you will get somebody who will give, I got these challenges I was using a stove or I lost my baby. But since then this very mother has never stopped using it…..”*(FGD healthcare worker).

#### Reflective motivation: Healthcare workers seen as credible role models.

Healthcare workers thought that pregnant women would be willing to adopt health education messages related to reducing exposure to HAP cleaner fuel alternatives following health education. This was because pregnant women looked up to healthcare workers as role models who were reliable and trustworthy sources of information. Furthermore, the need for positive birth outcomes were thought to motivate adoption of health behaviors. The health education was thought to cause some level of impact among pregnant women depending on their socio-economic status. This was thought to range from modification of the environment, use of curated wood/improved stoves to use of cleaner fuels.

*“…they have so much trust in the health workers. What a health worker tells them they always want to follow them because they think it is coming from an informed point of view. So, I am sure they are going to be so receptive because one there is no mother who would want a bad outcome for the baby….due to something that can be changed..”* (KII healthcare worker).

*“….if the health worker brings it well they will understand …..probably they will think of ways of using or maybe having a better kind of cooking kitchen somewhere but also I know that they can also improve on their materials they use for cooking”* (KII healthcare worker).

#### Automatic motivation to reduce adverse birth outcomes caused by HAP.

Healthcare workers would be motivated to provide health education on HAP as they had intrinsic motivation to reduce adverse birth outcomes including those due to HAP. Following the health education, midwives wished that pregnant women would embrace adoption of positive behavioral changes and make healthy tradeoffs like use of cleaner fuels. The health education was also perceived to arouse demand for cleaner fuels and measures to reduce exposure to HAP, while antenatal health education was seen to raise the awareness and risk perception regarding the burden of using polluting fuels.

*“We would wish to see mothers take up fuels which are safest to their lives. They should understand the cost of using cheaper but unsafe fuels versus expensive but safer fuels to their health.”* (FGD healthcare worker).

*“…I want to see that it [low birthweight] has reduced and then at least people are informed such that when you ask them they know that it is not the cigarette alone even this other smoke can cause problems”* (KII healthcare worker).

### Theme 3: What participants think should be done to enable HAP to be incorporated into routine ANC (opportunity)

Opportunity describes external factors beyond one’s control which influence uptake of a particular behaviors [23]. Participants suggested that training healthcare workers about HAP, involving healthcare workers when integrating HAP into ANC, using innovative teaching methods, customizing the current antenatal care services to include HAP as one of the screening questions, and motivating the healthcare workers involved in the health education would enable integration of HAP into routine antenatal care.

#### Train midwives to health educate about HAP.

When asked what would be required to make HAP a routine topic for health education, participants thought that the starting point would be training midwives about what HAP is, its mitigation measures, and teaching methods of how to educate pregnant women regarding HAP. Health education would help to raise awareness of healthcare workers, generate their interest and willingness to incorporate HAP as a topic for health education. The trainings could be through conducting workshops, and support supervision to enable them conduct the health education talks. Topics such nutrition were routinely taught to pregnant women because of the ongoing training workshops from development partners, the continuous support supervision and monitoring [20]. Training midwives would enable midwives to appreciate the impact that HAP has on pregnancy and birth outcomes and consequently incorporate it into routine health education talks.

*“….a big turning point is when someone values or knows the burden of something, that is when they will appreciate and know it is serious. So, …sensitizing the health workers about this burden and to make them really appreciate it is one point we should consider”* (KII healthcare worker)

*“We have to keep on making seasonal evaluation of how looking at the health education talks they have given….”* (KII healthcare worker)

#### Involve healthcare workers when integrating HAP into ANC.

In addition, involving healthcare workers engaged in health education talks was thought to promote successful adoption of HAP as a routine topic for health education. Pregnant women were mostly passive recipients of health education who had little or no preferences on the content of health education. Furthermore, involving the healthcare workers were thought to promote successful integration of HAP into the clinical care guidelines.

*“We need to involve the health workers as much as possible….but also we need to talk to the health workers since they are the ones who health educate. They are the ones who decide the topic… Because the participants, the mothers who will be coming for antenatal they have no choice other than to listen.* (KII healthcare worker)

#### Establishing partnerships with the various stakeholders was thought to be paramount.

*“I also believe we need partnerships to implement this, we cannot just implement it as an individual,…, we need partnerships probably…, with the hospital, with probably some other implementing partners”* (KII healthcare worker)

#### Use innovative teaching methods to inform pregnant women about HAP.

Pregnant women were perceived to have a short attention span, feel bored and detached from the health education sessions that take a long period. Therefore, devising innovative methods for health education was suggested to capture the attention of pregnant women, generate interest in the topic but also to facilitate midwives in conducting the health education talk. This would be through use of visual/teaching aids, pinning information and communication materials in the antenatal clinic waiting area, use of short videos and storytelling.

*“*… *We need to …..have teaching aids for household* [air pollution] *and the teaching aid could start with a story…….then they will start getting interested and trust me such a teaching method will capture them, will keep them hooked up for most of the session and they learn very well….. there is also an option of maybe using a video…”* (KII healthcare worker)

#### Include prompting questions about HAP on antenatal card.

Participants suggested that HAP should be included as part of standard care. This would be through introducing questions that assess use of polluting cooking fuels into health charts including antenatal cards, health information management systems, and hospital register. This would help remind the healthcare workers to talk about HAP but also ensure that it becomes the norm. Healthcare workers opined that there was a need to redirect attention from assessing practices like cigarette smoking which were uncommon among pregnant women to assessing common practices such as exposure to polluting cooking fuels.

*“Another thing that can be done…..if it is part of the package that is given in antenatal, ….maybe if we had a provision where we ask about that, the cooking fuel that we use, then from there if you identify, then you are able to educate or counsel the client about the dangers or the advantages of using that specific fuel”* (KII healthcare worker).

*“…We have a list of health education topics. So, this item should also be included on the list of health education talks.”* (FGD healthcare worker).

*“When you are taking the history of mother, like are you smoking? Really is that a question we can ask African Ugandan mothers?... there are no pregnant women here smoking… So, the smoke we shall[should] be asking is about the firewood…”* (KII healthcare worker).

#### Motivate healthcare workers involved in health education.

Although healthcare workers were motivated by desire for positive pregnancy and birth outcomes, use of incentives to create extrinsic motivation was reported to promote successful adoption of HAP in the earlier stages of its implementation. The extra motivation would remedy perception of HAP as extra workload for the midwives.

*“Some of them sometimes have that feeling of this is more work you are giving me…. So we need….some sort of motivation to simplify this. But if not, only the motivation can come there for the first few weeks when they have taken it up well, we phase it out gradually to make it sustainable that they will keep on teaching even without the motivation”* (KII healthcare worker).

## Discussion

The study explored perceptions of what was needed to make HAP a routine topic for health education during antenatal care. Our study noted reasons why HAP was not a topic for health education during antenatal care but it also identified feasible and practical measures needed to make HAP a routine topic for health education. HAP was seen as an acceptable topic for education by both the midwives and pregnant women, while healthcare workers suggested a gradual approach to promote short-term interventions during antenatal health education. The healthcare workers emphasised the importance of promoting adoption of shorter-term interventions given the socio-economic constraints that affected adoption of cleaner fuels. HAP education in antenatal care may play a role in promoting attainment of sustainable development goals including reducing illnesses and deaths from HAP and creating demand to increase adoption of cleaner fuel alternatives [27].

In Uganda, health education during antenatal care is currently focused on promoting adoption of healthy behaviors including prevention measures for HIV/AIDS, malaria and malnutrition [13,16,20]. Despite HAP being a major risk factor for adverse pregnancy outcomes [5], pregnant women, in our setting, do not receive health education regarding HAP. Limited knowledge among pregnant women regarding the effects of HAP accounts for one of the reasons for the continued use of polluting fuels [12]. In a setting like ours where 97% of pregnant women rely on polluting cooking fuels [2], health education during the critical stage of pregnancy could serve to help transition pregnant women from the precontemplation to contemplation stage to consider reducing exposure to HAP [28]. Beyond the superficial complaints of workload, the lack of capacity among healthcare workers was perceived to greatly account for the lack of education on the topic. Healthcare workers prioritized other areas for education which were deemed to have a major direct effect on adverse pregnancy outcomes, while topics like HIV dominated sessions for health education because of financial support, numerous incentives and training opportunities available for healthcare workers. Funding and providing support supervision for midwives could enable prioritization of HAP as a topical area for health education during antenatal care.

While healthcare workers received pre-service training on cigarette smoking, a practice which was not common among pregnant women in our setting, healthcare workers were not trained to provide education on HAP. Consequently, healthcare workers had limited knowledge regarding HAP which underscores the critical role of capacity building in integration of HAP as topic for antenatal health education. Like in our study, midwives were hesitant to provide education for fear that they would not be able to answer certain critical questions that pregnant women may raise during the talk [29]. Training healthcare workers would enable them to appreciate the burden, and the need to develop the capacity to educate pregnant women regarding HAP. Policies and clinical guidelines in Uganda need to be revised to reflect the relatively the high burden of HAP from polluting cooking fuels than from cigarette smoking. Considerations should be made to routinely assess HAP as one of the risk factors for adverse pregnancy outcomes through deliberate effort to integrate it in the antenatal care services, and health management information systems.

Although informing pregnant women regarding the effects of cooking fuels was deemed to be appropriate and acceptable, health education was seen to create a state of paralysis resulting from cognitive dissonance related to inability to change. Shorter-term interventions (e.g., outdoor cooking) were thought to be feasible for pregnant women who could not adopt cleaner fuels. In Uganda, pregnant women were able to make behavioral changes to avoid HAP, improve ventilation and cooking outdoors following a midwife-led health education during antenatal care [21] suggesting the feasibility of implementing shorter-term interventions. While current emphasis is on adoption of cleaner fuel alternatives albeit with much success [7], shorter-term harm reduction interventions should not be ignored [8]. Although exposure to HAP from short-term interventions often remain above the World Health Organization Indoor Air Quality guidelines, they have been shown to have some health benefits [8] which underscores the need to promote their use as households transition to cleaner fuels. Continuously exposing pregnant women to health education on HAP could create awareness, and empower adoption of behavioral changes that reduce exposure to HAP.

Consistent with our study, previous studies have highlighted socio-cultural and socio-economic factors including financial constraints, beliefs and attitudes against cleaner fuels as the major barriers to behavioral change [7,12,30,31]. Interventions which focused on behavioral change without providing pragmatic support to cater for cost constraints did not significantly reduce HAP [32]. The HAPIN trial showed a high fidelity and exclusive use of cleaner fuels following provision of free and accessible LPG stoves though it was in the context of experimental setting without long-term follow-up [33], indicating importance of removing financial challenges in promoting adoption of cleaner fuels [33]. Providing cleaner stoves on credit or through use of affordable and flexible payment mechanisms can offer feasible solutions to financial constraints hindering accessibility of cleaner fuels [34]. Although some of the changes to reduce exposure to HAP were not costly, attitudes and beliefs that smell from LPG was harmful, hearsay stories of LPG explosion from improper use were seen to hinder adoption of cleaner fuels alternatives, a finding which was consistent with previous studies [12,21]. Concerns of security, housing structure and weather conditions were seen to affect adoption of practical solutions such as outdoor cooking. Pragmatic health education, however, can provide households with practical solutions to problems of making adjustments in their environment including ensuring ventilation, cooking outdoors, and keeping children away from cooking place [21]. Previous studies have found visual messages effective in addressing misconceptions that households have regarding safety fears and reluctance to use LPG [35,36].

The study provides insight of both healthcare professionals and pregnant women regarding what to consider to enable a successful implementation of health education program on HAP. The study findings are grounded in the data. The findings may be limited by social desirability bias. The difficulty in establishing criteria to recruit women who could provide rich data may also have limited the nature of interview or responses. The social hierarchy between the interviewers and the pregnant women may have affected the nature of responses. The study has not described the socio-demographic of the participants which may affect the transferability of the findings.

## Conclusion

Our study noted that health education regarding reducing exposure to HAP during antenatal care was not done because of individual, attitudinal and healthcare system factors. Training healthcare workers to health educate on HAP, using innovative teaching methods, motivating and involving healthcare workers would be critical in scaling up health education program during antenatal care. The gradual approach to advice pregnant women to adopt shorter-term interventions to mitigate exposure to HAP was thought to be more practical, feasible and likely to be embraced by pregnant women. Cost implications were the major barrier to behavioral change. Integrating health education on HAP during antenatal care, when pregnant women are willing to adopt healthy behaviors, can empower pregnant women with information, offer solutions to reduce exposure to HAP, and create demand for cleaner fuel alternatives.

## Supporting information

S1 Text Inclusivity in global research. (DOCX)

S1 Table COM-B framework to understand what was needed to make HAP a routine topic for ANC health education. (TIF)

S1 Data FGD with healthcare providers. (PDF)
