# Supplementary figures and images for: Perceptions regarding making household air pollution a routine topic for health education during antenatal care: A qualitative study in Eastern Uganda
Source: PLOS Glob Public Health. 2025 Sep 15;5(9):e0003973. doi: 10.1371/journal.pgph.0003973 (PMC12435645; doi:10.1371/journal.pgph.0003973)

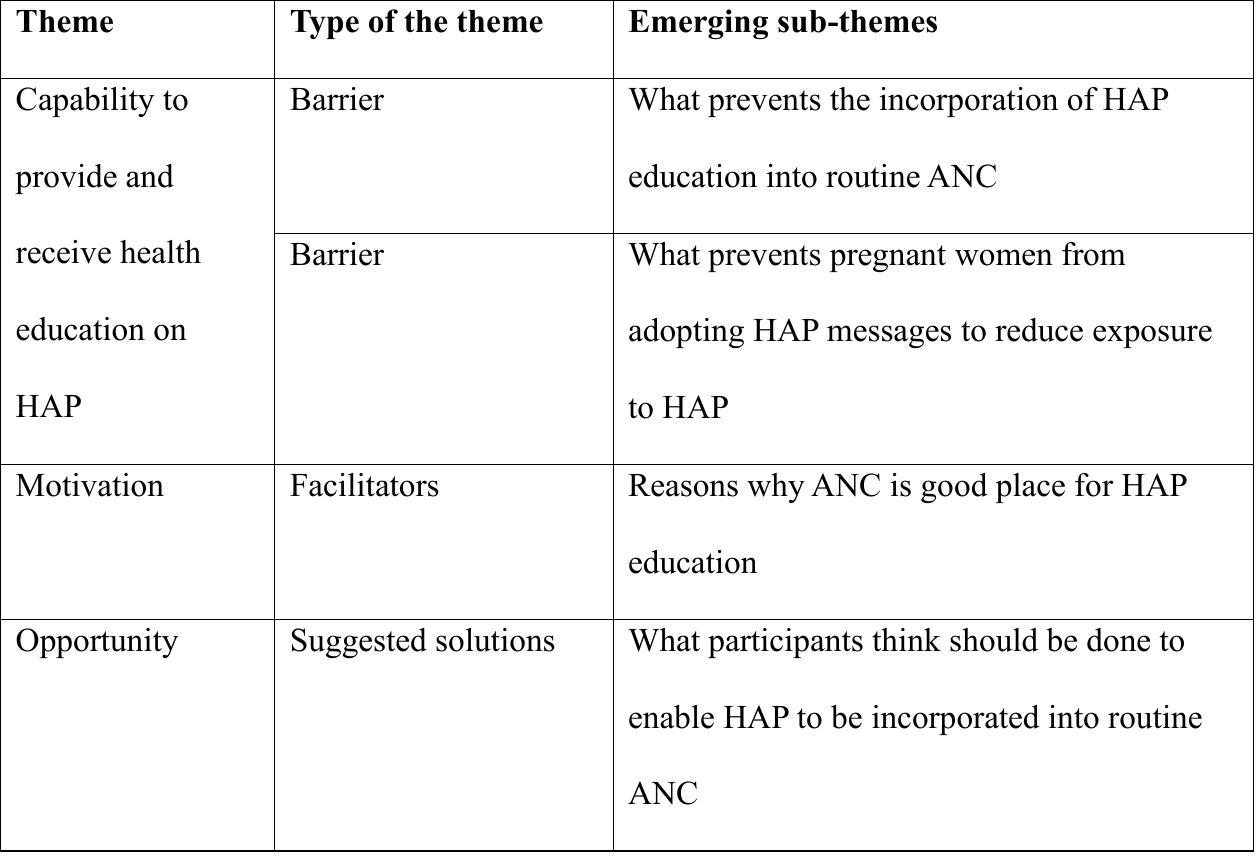

Supplement: S1 Table — (TIF) [file pgph.0003973.s002.tif]
