# Supplementary material for: Perceptions regarding making household air pollution a routine topic for health education during antenatal care: A qualitative study in Eastern Uganda
Source: PLOS Glob Public Health. 2025 Sep 15;5(9):e0003973. doi: 10.1371/journal.pgph.0003973 (PMC12435645; doi:10.1371/journal.pgph.0003973)
Supplement: S1 Data — (PDF) [file pgph.0003973.s003.pdf]

FGD with Healthcare providers

Interviewer: If I am to pose the first question to us here, what comes to our minds when someone says house hold air pollution (how do you perceive it when someone tells you there is household air pollution)?

Respondent: Thank you, to me when I hear about household air pollution, I will think of things that we use in homes in form of our daily basic needs but at the end of the day what we use affects our health without a notice especially when the environment is not very aerated. For example the fuels we use like charcoals, you are in the same room and you are using charcoal for cooking, that smoke there the children are inhaling even yourself you are inhaling the smoke. In the long run that smoke can cause some end health with time without your notice and that is the commonest type of fuel that the community tends to use. So that is what I think as me.

Respondent: Well, in addition, I am not so much different from what sister has talked about, but what I know is the opposite of fresh air. It can be anything that makes fresh air not to be good in the house.

Interviewer: Thank you, so we are majorly concentrating on what we use at home for cooking, not dust. So do you think there is any effect of the things we are using at home say electricity, gas, stove, firewood, cookers to our mothers?

Respondent: What I can say, as we use these charcoals, gas especially the pregnant mothers and the baby may have less oxygen supply which may lead to the retardation. The growth of the baby, the baby may not grow well, this baby may develop with some complications. Because this baby has got some problems during intrauterine life, the baby may be born with some complications like we are getting very many babies born with the holes in the heart, it might be one of the causes because I think no results have been done about it. If you are doing this results may be something was done before but still the problem is not going away. Now by doing your results may be looking for another alternative of what could be done to prevent that thing from increasing.

Interviewer: You talked about charcoal, could there be other fuels that we use that are harmful to pregnant mothers and baby?

Respondent: Gas is harmful because you may open it and delay to put the light on, that smell which is coming can cause problems.

Interviewer: Most of us who are there are ….., what is being done currently to reduce this effect in our antenatal care?

Respondent: I would say when we discuss or give a talk, we kindly encourage them to have well ventilated house because there is no way they are going to avoid to use it. They have to but just have to make sure there is enough ventilation to the person using it especially a pregnant mother.

Respondent: Sometimes mothers, they share or somebody might have rented a house whereby she is using that same room with stove for cooking than putting outside, so we encourage them to at least separate it, to put stove outside when it is not raining or else you can build up a separate kitchen where you can use stove or fire wood.

Then as ….mentioned of the fetus can get infected when the mother has inhaled some smoke. Sometimes it depends like when this is a mother with some syphilis, placenta is weak, the smoke can go through from the placenta to the fetus, but if the barrier is ok, according to me the fetus may not get effect.

Respondent: I think many times we tell them to avoid cooking at night using a charcoal stove because when you use it, majority end up lacking oxygen or may lead to death of both mother and the baby.

Interviewer: Could you mention some of the barriers that can stop us from talking to these mothers?

Respondent: Some barriers we find when we are giving a talk is language. You may really want to go in depth to explore the information to the mother but you find that she does not pick the language or you don't the language that mother speaks. So that is the most barrier thing that happens between us the health educator and the clients.

Then also time management of mothers coming in. Most mothers when you listen to their conversations they say they are bored with health education, so they want to come late when we have finished health talks and we have started carrying out examinations. They just say for me I will go at this time when they have finished talking their things then for me I just go they examine me and then I come back quickly. So also time has really become a barrier, however much you tell them to come early for them they don't value health talks so much, they value to be checked and go away.

Then may be sometimes shortage of man power, you really want to have time to talk one by one but when you are alone you find yourself in early, you have a lot of things to do, so it also becomes a barrier for the talk.

Respondent: Also to add on that, some of the barrier I can mention about is if you see the mother you are giving health education, this is a mother that is not feeling ok that also can contribute. I think like when you don't give respect to these mothers like you start giving health education like you woman ….., so they can lose moral of listening to whatever you are talking to them.

Respondent: Also lack of teaching aids like you may have posters in which there are those things like a mother cooking using a charcoal stove, fire wood, gas, stove, so those things should be there to demonstrate. When you are teaching you demonstrate this is a stove, this is a gas for someone to understand what you are saying.

Respondent: Thank you for all your presentations, apart from time management as … has said, staffing is a problem, then inadequate explanation to the mothers can even become a barrier, then late reporting affects both the mother and the….. When the … comes late, the information given may not be adequate and when the mother comes late due to some barriers/ problems like weather, distance, transport are all problems. Then lack of visual aids, some of them want to see physically.

Interviewer: Just to add on the very points, what do you think if we have such barriers, what could be some of our enablers/ promoters that we have at our respective workplaces that would enable us to integrate this education in our daily duties?

Respondent: Like when we have shortage of labor, what enables is presence of students, when these students are there they can really help us, then have time and give a health talk. When they are there, there are small things they can do then for you get time to give a talk or if you can discuss with them, you can help them also to say something as we discuss with mothers.

Also as talked of time, we have to improve on time and we have to keep talking to the mothers. The way we bring out the topic I think if it is interesting, these mothers will pick interest, but if we don't bring it out very well they get bored and they don't pick interest.

Then may be as usual we have been involving the men/ husbands for the issues like transport, to be supported behind so that they can be able to reach the facility as fast as possible.

Respondent: We can give more knowledge and skills to the community through radio talk shows because this is a new topic. Then outreaches and fliers, we can prepare fliers for any new mother who comes should be given that flier has to go with it at home. Then even home visit by a midwife, you go and do supervision on how their condition is.

Interviewer: So with that as …., do you think there is any need or is it appropriate to integrate this household air pollution education in our counseling sessions?

Respondent: I think there is need for integration because we see that air pollution is a life threatening thing to the mother and the baby, so when we integrate it to these mothers who come for antenatal, they may be able to go and take precautions on how they use fuels which lead to pollution. So I think it is a great need for that.

Interviewer: I there any other person with additions.

Respondents: All silent……………………………………………………………………

Interviewer: If we really see that there is need to integrate this health education in our counseling sessions, what does it really cost us/ demand of us as midwives to implement this health education to our mothers?

Respondent: If we say it has been integrated, it means it is going to be carried out as any other activity that takes place on a daily basis. The same …., who has been giving the other health talks and counseling, is the same … that is going to do this following the schedule that has been made. The same time that you have been carrying on health talks and counseling, it is the same time that you have to give this health talk to mothers about fuel pollution. Basically it does not cost anything other than the usual commitment that you have been having to deliver it.

Respondent: For me I think it is going to cost us to convince these mothers that it has side effects because to convince them to accept remember they have been using ever since now for you here you are coming up and saying it is risky, it does this and the other. It is going to cost us some time to convince these ladies that really whatever they are using at home has some side effects. But anyway they will come up and ask that now what do you want me to use or these precautions may not work for me, I am renting a small house I have to cook inside when it is raining. Such questions are going to come out, so it is going to cost us time to answer those difficult questions. They have been using and you say they are risky to you and your baby and then she is like is there any option to use others other than what I have been using.

Respondent: According to what I feel I know they will accept it but now the challenge is how am I going to stop using it, even among the mothers the evident are there, you will get somebody who will give, I got these challenges I was using a stove or I lost my baby. But since then this very mother has never stopped using it, she is still using the method of cooking. She lost the baby because of the fuel but now stopping it is the problem.

Interviewer: As …. having these discussions held in our daily health educations or counseling sessions that we conduct at the wards, which interventions can we have to ensure regular/ daily/ continuous health educations go on in the wards or our respective work places regarding this topic?

Respondent: Of course previously we have been having health education talks; we have a list of health education topics. So this item should also be included on the list of health education talks.

Respondent: To add on what …. has said I think it is going to be a continuous conversation/ topic. You interact; it can be one on one or even in a group. I can have a pregnant mother either related or not, so in our talks I chip in this topic. So that is what I think we can do.

Respondent: We can also have posters, we can also put it somewhere for people who are able to read or can put it in several languages like….. and we put them in some specific areas where people can see it and read.

Respondent: You can arrange days then you go to the radio and talk in any language people can understand and then for us like antenatal with ongoing health education, it depends on the day we give health education on the ward burden of this smoke, it can affect the mother and the baby.

Interviewer: As …., we look at the future of our mothers, regarding household air pollution effect to them, what would you want to see change regarding the education of these mothers on household air pollution (What are some of those key things you think we need to change in future as we educate the mothers)?

Respondent: I think one of the areas is on the building plan; the house should have enough ventilation

Respondent: We would like to see that these mothers take it positively in a way that someone can be able to choose what to use for example the paraffin stove, someone can choose to use. This one affects someone so much than may be gas or charcoal, so what I am trying to say is we want to see a positive change towards our topic.

Then also we want to discourage them to cook under locked for example at night because we have heard people suffocate at night when all rooms are closed. So we want to see these mothers accept that it is bad to cook when the house is closed.

Respondent: I would say we would wish to see mothers take up fuels which are safest to their lives. They should understand the cost of using cheaper but unsafe fuels verses expensive but safer fuels to their health. Me I would look at that or expect that as a change.

Interviewer: …. talked about changing the building plan and fuel plans, if you put yourself in this pregnant woman's shoes, are we willing to change from charcoal or firewood maybe to gas and how appropriate or feasible is it with the economic status of our mothers that come to our hospitals?

Respondent: According to me, knowledge is power, once these mothers are given this knowledge, they will not change as per say I am going to change from charcoal to the fuel which they cannot afford because remember they are economically handicapped, there is poverty running but at least if she given information, she is able to change in form of environment; if she has been cooking from the same room where she is sleeping, with knowledge and power they are able to put up a small shade that we cook from the other side which is a bit aerated and then we sleep this side. I think that is what may benefit them but if we say that we switch off from charcoal to fuel with this economic status, I think it will be gradual, not so fast.

Interviewer: If they take this information to the husbands, do you think there is need to also incorporate the husbands or we just need to talk to the mothers/ will there be change if we incorporate also the husband?

Respondent: In our African setting, the husbands are the key to the success of everything. As we plan this to involve antenatal mothers, I think it is good to involve even the spouses because they are the key source of income, head of the families. What is positive they will receive and share how do we prevent this, nobody wants a sick child. Our child is to have a health mother to give

us a health child for the future. So I believe when we involve the men it will yield more fruits in whatever we are planning as per this pollution.

Interviewer: We want to conclude with our concluding remarks like we would want to list the enablers, the messages that will enable these ladies change from maybe cooking fro same sleeping room/ what will enable this woman to easily pick up your message and take it home other than you telling me that you stop using stove. How soft can we midwives be if we are to change these mindsets of a pregnant woman?

Respondent: I think every health talk that we give has advantages and there are qualities of a health educator. A health educator should keep time, should be at the same level with the people you are communicating to and should have a target audience that you are looking for. And a health talk should always be precise to the point, in other words your goals when you are giving a health talk should be SMART (Specific, Measurable, Attainable, Realistic and Time bound). So you be audible enough, be straight to the point and let it carry value. Like I come and say this topic is going to take us 20 minutes, let it be 20 minutes and whatever the person has grasped will not come out of that person though she may not have immediate change but with time she may think of making a change because you have empowered.

Respondent: It will be a bit challenging because when we say we build a house, this is a mother, she rents, now where is she going to build another house.

Secondly may be she is a working class, ….talked of discouraging to cook a night, may ne she is a working class, she comes at night so maybe she can use a stove or something and there is no way to avoid using charcoal when inside, may e it is a rainy season. So it is a bit challenging according to me.

Interviewer: …. has mentioned of some barriers, could there be other barriers because when you are teaching you can give a barrier and say how can we solve this like tell them I know you are renting but we can do like this?

Respondent: For me one thing I know about health talks is your work is to give knowledge, let the person get the knowledge and take it home and your know Rome was not built in one day, with time as we continue talking, giving the disadvantages of cooking in the house they will pick the message and there will be a positive change, because we are not just going to say today do this and they do, no it will take us time, we may have to or if there is any other health talk you are giving we chip in this one every day, it will cause some change.

Interviewer: I think we have finished, any other view or anything you want to mention?

Respondent: Among the agents of air pollution, is there any which is safe? We have mentioned of charcoal, firewood, gas, stove, is there any which does not have risks to human health?

Transcripts with women

As I said, I will be recording, and number one is

Tell me your experience about the talk that we have just had? The health talk about biomass.

Participant: even in Luganda,

Interviewer: Yes, it's fine. Just tell your experience

Participant: ok, we have been in ANC, and we got some healthcare workers who taught us about biomass and the experience. I got is that while we are cooking while using firewood, we should be in an open place or let's get someone to help, not to be taking in that smoke so much because it has the effect we can get as mothers and even the unborn babies. One of them is bleeding and not breathing well and maybe premature birth. So, I have learnt personally that I should not stay near the cooking place/ smoke as pregnant lady

Interviewer: thank you any other experience?

Participant: Hmmm we have been learning about biomass and the healthcare worker explained to us that we pregnant mothers should not be close to that smoke.

Interviewer: This is a discussion. So, when you're talking you make sure that even the friends hear and they don't repeat

Pat2: okay, that when making charcoal, lighting the stove, we should light from outside such that we don't get that smoke as pregnant mothers. If you don't, you get a problem as a pregnant mother like bad breathing like that and it lead to some of the risks like premature birth, and also another one, bad breathing hmmmm that's what I got.

Interviewer: ok thank you,

Participant: Hmmm thank you I came to ANC and I have learnt many things about biomass on which some, I was not even knowing and I was a victim some time to use some firewood but my coming today I have learned a lot of things. They taught us that maybe when you're burning things from outside, you have not to be near that gas because those plastics, they have very many things that can bring bad smoke. That smoke can enter us, through us and can bring for us problems such as for us a mother, you can easily get high blood pressure, some can go to the unborn child and affect the child to be delivered when is weighing less grams and not well matured. These can affect the child. That is what I have learned and it has helped so much

Interviewer: Ok anything more. Ok the thing is, now these proceeding questions whether you came late or earlier you can answer. Hmmm what do we use mostly for cooking at home? I think this is personal. What do you use mostly?

Participant: For me I came late.

Interview: Is it charcoal, firewood or?

Participant: Charcoal

Participant: For me, I use charcoal sometimes firewood, in the evening sometimes I mix firewood and charcoal. Fire wood charcoal, stove. Sometimes I can use when it's sunny, I use firewood. When it's raining and it's late, I can use charcoal. In the morning I use stove.

Participant: Me, I use charcoal

Participant: Me, I use charcoal and firewood.

Interviewer: So, there is no one using electricity?

Participants: No

Interviewer: What of gas, bio gas

Participants: No, we don't have

Interviewer: Maybe this mama of the dinning

Interviewer: So, apart from the few that you have listed that it can cause bleeding, low birthweight, premature birth, are there other effects of using charcoal and fire wood. Because it's like for us we shall be using charcoal and firewood?

Participants: Apart from what?

Interviewer: Apart from the effects that you mentioned about using charcoal and firewood, you talked about premature birth, low birthweight, bad breathing and bleeding. Are there other effects that these fuels can bring to us?

Participant: Now healthcare worker, like to us, when we are cooking inside as pregnant mothers when our windows are not open, can't we faint?

interviewer: Yaaa even us who are not pregnant can faint especially when cooking beans. What of this side? Another effect please?

Participant: Maybe another effect is when you're using like charcoal in the house and every place is closed. It can add this carbon monoxide gas and it can lead to death.

Interviewer: Yaaa that's a very good point even to us who cook beans and we don't open windows. It can affect us. Even to us who have dinning and we don't open windows. It greatly affects you.So, do you think these health talks are helpful and beneficial?

Participants: Yes

Participant: We learned very many things

Interviewer: You learned very many things? Is there anything that you would like to talk about? Because I see most of us are using charcoal, fire wood, given the talk, are you willing to change?

Participant: I would really be willing to change but it depends. You know gas is very costly and you most of us cannot manage to get it. So, we are moving on with what we can manage

Interviewer: Like we what can you manage

Participant: For me I can manage charcoal and firewood

Interviewer: But you see with charcoal. There are always two types of stoves, there those one made of iron sheets and those made with some sand inside and small space is left in the middle for charcoal… those improved stoves

Participant: Energy saver stoves

Interviewer: Yes, that one with a small hole, you know that thing. Who is using that one? Because this one is a bit ok. It produces less smoke. It produces much heat after heating its walls. But now my question is, when using these cooking stoves which measures do you take to reduce exposure to the cooking fuels? Some people will say, for me I put the stove outside

Participant: When I ma using the stove I light it from outside and when its gets light I put it inside.

Interviewer: But are you willing to be taught these things when you come for antenatal? Because some women when they come they first sit outside wait for the health talk to end before entering?

Participants: No. They first wait for the cleaner to finish, and also for the health workers to prepare. We wait for them to call us. So, we wait from outside.

Interviewer: Ok, now, here we are, I asked the question you didn't answer. The question is, what changes are you going to make at home to reduce exposure to cooking fuels?

Participant: Like my side I will start using dry firewood. So that the smoke in the chicken is reduced. I will also make sure that I use a proper stove that produces less smoke and also put it outside.

Participant: Me I will make sure I light my stove from outside then when it's ready that is when I enter it inside the house and I start cooking and the windows should be open

Participant: Like me, I have a helper. I will make sure she makes it for me and when it's ready I will begin cooking

Interviewer: So, there is another option of getting a maid. What if she is also pregnant, uhmm?

Participant: Yes, if I have a pregnant maid. I make sure that I help her. We light the stove from outside such that we reduce the exposure to the fuels and then when it's ready then we can enter it. And then if we are using firewood, I will make sure the firewood is dry and then the windows should be open …yaaa

Interviewer: But what do we use for lighting at home. Because we are looking at cooking fuels not forgetting the candle. The kerosene lamps.

Participant: Wait. For me, I will make sure that the windows are open and I use dry fire wood

Interviewer: Ahaaaa, what measures are you going to take to reduce exposure to the smoke?

Participant: Pardon. It's a pass, for me I can't cook

Interviewer: You have a maid.

Participant: Yes

Interviewer: But you have been cooking

Participant: Maybe we stop using polythene bags

Interviewer: We have been moving in communities but the there is a lady who has been distilling alcohol using shoes, like she gets those slippers, plastics and then uses them for cooking. So, there are also women who are cooking using those materials. There women who are cooking using coffee husks and timber husks so if we are using these. Another option is to tell our husbands to put up small kitchen and they put a log chimney so that the smoke gets out through that chimney. Hmmm lastly, which barriers are we facing in implementing these solutions that you have talked about. Like when you're cooking while windows are open. You may say, that mosquitoes can enter. Ok like getting gas, which barriers are getting in implementing these solutions?

Participants: Pardon

Interviewer: We have said that we shall be opening windows, cooking from outside, using safer fuels among others but which problems or what hinders us from doing all these things?

Participant: The challenges we get after doing the right thing, maybe I can open the window and wind is too much and it can put my fire off that can force me to close the window. Secondly maybe what can be there, the security is not there, the place is no secure. Maybe I can close the window in order to be secure.

Participant: Maybe another barrier, for example maybe when I am in a rental, the place is very small. You can't say that let me put here my stove and wait. For example, the place is not safe for

cooking. So, you decide to make it in from inside. They can even steal your food. So, this makes you cook from inside.

Interviewer: Hmm what about these modernized methods like using gas? Gas rarely brings out smoke but what prevents us from using gas?

Participant: Hmmmm very expensive. The economy

Interviewer: But do you have power at home?

Participant: But healthcare worker, I maybe renting then affording gas! And now if land lord has put there Yaka [electric bills]and then you start cooking using power.

Participant: For me, I even don't use electricity. I have my solar but now electricity. Electricity is very expensive. Because if you try to use like a percolator, just minutes, the electricity unit is gone. So, times situations catches us up and we have no other option.

Interviewer: Ok hmmm then gas, does it have any problems? Do you find any challenges with using gas other than being expensive?

Participant: Yes, there are challenges like…. I have never used but I hear if you maybe make any slight mistake, it will burn the house or it will burn the children and if you leave it with the children, they can play with it and then they get burnt. So, me personally I fear gas.

Participant: Sometimes, though I have experienced it, I have ever used it. When the gas is over, there is some gas that remains inside, so sometimes you say that let me leave it there and use charcoal, that last gas, a child can put it on which can bring bad smell or gets fire. And …(ignoring gestures)

Participant: I have never used electricity and gas.

Interviewer: Biogas?

Participant: have never used

Interviewer: Members thank you for the discussion and is there anyone who has a question?

Participants: No

Interviewer: Ok, Thank you for your time

Key informant interviewer with a healthcare worker

Interviewer: You are welcome to this interview; the purpose is we want to understand how we can integrate health education about effects of cooking fuels in routine antenatal care so that it becomes a routine topic for health education. Yes, that what we want to understand. So, we can start with what is currently being done in antenatal care on health education, do you think this topic is one of their routine topics for health education talks?

Respondent: I think in antenatal , I have not attended so many of the health education talks in antenatal but the few I have attended to basically they are talking about the risks that are related to the pregnancy, they also educate them about family planning, they also educate them maybe in case of any danger signs, how they can identify and come to the hospital, then for those who about to reach term they talk to them about the delivery plan, that is what I have noticed, I have not specifically heard them talking about the cooking fuels.

Interviewer: What do you think are some of the reasons why it is not part of the topics that you have mentioned for health education?

Respondent: I would think maybe why it is not because some of those different fuels that our clients use sometimes depend on the situation I think they have. If you can afford gas have it if you cannot afford you find they are using charcoal or they are using firewood. Two, we may also under look it and you may think maybe the health workers under look it may be thinking that it is not a high risk, that is just a thought.

Interviewer: So, if you want to make this also to be a priority topic for health education, what do you think needs to be done?

Respondent: So, what needs to be done if you want to make it a topic, one is to meet the health workers who work in antenatal and maybe we educate them about the dangers or the advantages of using specific cooking fuels or the dangers of using certain cooking fuels so that they are also informed, health workers are first informed before they start giving health education to the mothers.

Interviewer: So, your point of training the health workers first.

Respondent: Yes, to inform them about ……. Because I am just giving myself as an example if when I am giving health education it may not come out easily like I have told because most of the other things sometimes are not avoidable, maybe the person cannot afford what is ideal.

Interviewer: Why would you think that we don’t see it like the way we see the danger signs of pregnancy. Why is it being under looked as an area for health education?

Respondent: I think the challenge that we see maybe in the department we tend to focus so much on the pregnancy so when the mother even when we are doing antenatal, you are not even asking

these other things even on antenatal card it is not even there, so we tend to focus so much on the pregnancy and maybe the things that are on the card, are you using mosquito net, have you taken fansidar. Then two because of maybe numbers sometimes it is hard to talk about so many things because the clinics are always very busy.

Interviewer: So, do you think the midwives have the capacity with your experience working with them, do you think they have the capacity to make this a routine topic?

Respondent: Yes, they do have the capacity because they are the ones who do the health education and sometimes, they do with the students if we have the students on ward but still they are the ones who educate the students how to do the health education, rarely will you find maybe like a medical officer because when we come to the clinic we start the clinic right away, so health education is basically done by the midwives.

Interviewer: So, you talked about the aspect of training the midwives first regarding household air pollution, use of cooking fuels before they can cascade it to the women, what else do you think needs to be done if we want to make this a routine topic for health education during antenatal care sessions?

Respondent: So maybe another thing that can be done, I don't know maybe this may not be in the means of the study if it is part of the package that is given in antenatal, the package I mean like how you see on the antenatal card where they put mosquito net use and you are writing yes or no, maybe if we had a provision where we ask about that, the cooking fuel that we use, then from there if you identify then you are able to educate or counsel the client about the dangers or the advantages of using that specific fuel, but I don't know whether it is in the means…

Interviewer: Ok, it is a good point actually because it guides then when you have something like a checklist and say we have talked about anemia in pregnancy, I have talked about sleeping under mosquito net, I have talked about how to avoid smoke from cooking fuels, I think they would be able to remember it.

Respondent: Because most of the things that are missed during health education, so when a patient comes in antenatal room you are checking what is the date you write the date, how many weeks of amenorrhea, because you have like 70 people to see outside so it is hard for you to go through those different histories.

Interviewer: Then the other aspect of sustainability, you know sometimes when we come in especially when you are not part of the antenatal care setting, you go and train people and you tell them you start doing this, do you think it is feasible to make this a routine topic with whatever we are doing right now?

Respondent: Yes, I agree it is very feasible, maybe how we can also do it after we also need to get like some flip charts, we put them like in the antenatal so that if a woman comes, maybe has

also come with a husband they are able to see the dangers of using this fuel when we have the charts on the wall because sometimes you will come and do the health education, this lady goes home and tells the husband the healthcare worker has said we stop using stove yet in actual sense is not understanding but when he comes and you are able to see those things pinned on the walls, the clerkship rooms, they are able to keep communicating, so it means if that wall paper is on the wall for like 2 years it means you are communicating for a period of 2 years to the different clients.

Interviewer: Wawooh that is interesting, so the other thing is about the midwives because they are the ones who are going to do the health education talks, if we are to tell them that let us make this a routine topic, do you think they will find it acceptable or there will be some challenges with it?

Respondent: So, how they will find it acceptable is if they also understand why we want to make a routine topic, so that is why I told you we need to first tell them why, the advantages, the risks these different fields put to the mothers but if they do not understand the risks then it will be hard for them to accept it because you know like I told you when you are in antenatal you tend to talk about the pregnancy and this other maybe it tends to predispose to respiratory or what we tend to under look them.

Interviewer: So, emphasizing the effect it can have on the pregnancy.

Respondent: Yes, the effect it can have to the woman, and then if the midwife clearly understands those effects then they can also emphasize it to the mothers.

Interviewer: So, for now do you think our midwives are well versed with the effects that might come up with the use of cooking fuels?

Respondent: I am not sure, we haven't discussed.

Interviewer: Are there any cost implications if we are to make this program part of routine antenatal care, are there some costs that the hospital may incur?

Respondent: I think there will be no costs because if it is health education, we have been doing routine health education, may be if there are initial costs like what I have told you if we are to make like flip charts, they can be like only 4, the cost will only be at the start then the rest I would think there are no costs because we have been doing the health education, it is just to introduce this topic into the package we have been giving.

Interviewer: If we are able to provide all those materials, do you think that the midwives will be able to regularly do the health education talks?

Respondent: Yes, I would think so

Interviewer: Or there will be need for continuous mentorship or supervision.

Respondent: I would think they will regularly do it because you see these clinics unless maybe they have done like a reshuffle and the person who has been here has been taken and they have brought new ones then we take through the new ones then after I think they put it in the package because usually you find they are the same people who are there all the time, so if they are aware unless there is like a reshuffle and they have brought like new staffs.

Interviewer: So, we have been talking about effects that cooking fuels can have on pregnant mothers and you started by saying that right now we are not doing it, so what would you want to change in the future in regards to health education in antenatal care? What would you want to see in the health education sessions that we routinely give?

Respondent: Because when you clearly understand the effects the smoke would have because you almost be like a passive smoker on the pregnancy, even not necessarily the pregnancy, even o the health of the mother, of course they have the side effects, so to me as a health worker of course I would want to see a good outcome of both the baby and the mother so that the baby doesn't have any of the effects that are related to the fuels. So, when it is in the health education package it may change a lot to the mother, some mothers I think use some fuels when they do not know the effects to them yet they can actually afford the fuels that area bit safer and some of them maybe use them in closed doors because of the ignorance they do not know. So, we are health educating and tell you if you cannot afford this and you are using this make sure you are in an open space or make sure you are in a well-ventilated room, if you are in a single room make sure you maybe cook from outside, so just a few things to tell them ad inform them of the dangers even when they cannot afford to get the better one, but whatever they use you can tell them to use it effectively.

Interviewer: So, it is interesting that our mothers maybe ignorant and yet when they come to the health facilities we do not actually inform and so they go back the way they came. I think that is interesting on what you want to see change in the future. So, the last two questions I have, number one is about women and their families, if we are to make it a routine topic how do you think they will receive it?

Respondent: You know one thing that I have seen about clients or patients that come to the hospitals, they have so much trust in the health workers, what a health worker tells them they always want to follow them because they think it is coming from an informed point of view, so I am sure they are going to be so receptive because one there is no mother who would want a bad outcome for the baby, there is no mother who want to get an illness due to something that can be changed, so I would think the reception would be good. Then two sometimes just like I have told you there are few things that would maybe need to change for effective use, sometimes they may not need to incur any extra cost so it is just adjusting on the use.

Interviewer: I think you have mentioned some of those points about us telling others what to do to avoid the smoke, so are thee some barriers that might come out with them being able to put the messages that we give in antenatal in to practice?

Respondent: Yes, so the barriers one that affects us most is about the socioeconomic status, let us say may be to be specific on the fuel, you find someone is in a single room, that is where they cook from, that is where they have other children so the whole family is packed in that single room so that one can be a barrier because they cannot afford a better fuel that is more safe because maybe it is not affordable. I think basically the main barriers could be the socioeconomic status. Then two maybe if the client comes because sometimes we health educate, some of them are not even paying attention or some of them have not picked what exactly you told them especially being pregnant, they may just say aaaaah me I am pregnant how will this smoke reach the baby, some of them may not actually take in the information but the main barrier would be I think the socioeconomic status, even when they want to change you find now it is at night or it is raining, they are cooking from house shade so they are taking the stove inside the single room they are sleeping in, they are lighting those stoves for paraffin what are they called, it is what they use for light so they are inhaling those smokes.

Interviewer: It is interesting and any other enablers that might come that might make them be able to take up the messages that we give in antenatal, I know you talked about that they trust us as a reliable source of information, any other?

Respondent: Of course, the other thing because we usually tell them the package, we are giving we are saying at least 8 contacts, so if every time the mother comes if they missed it the first time at least they can get the second time so by the time they finish the 8 contacts, at least they are able to pick so continuous like where you said we need to sustain it so that every time you have contact with this mother, you are able to inform them about the fuels.

Interviewer: So now we have come to the end of our interview, if you have anything you want rise regarding what we have discussed about making this a routine topic for health education talk that we give, you are welcome.

Respondent: Thank you for having me, I think I don't have, we have discussed almost everything.

Interviewer: Ok, thank you.

Key informant with healthcare worker

Interviewer: So, you are welcome to this interview, we are trying to understand how we can make health education about effects of cooking fuels, you know when they cook the smoke can have an effect on pregnant women and unborn children. So, we want to see how we can make it a routine topic for antenatal care. Like the way we do for anemia n pregnancy, danger signs, preeclampsia, how we can make this one also a routine topic. So, let us start from the very beginning, what is currently done in antenatal care regarding health education about this subject?

Participant: Currently in antenatal care, health education takes place usually in the morning when almost all the mothers arrived. The midwives come and introduce themselves and then there is usually a topic for each and every day assigned. So, the topic can be there on HV/AIDS, topic can be there on nutrition, another day you find a topic on birth preparedness and then it is given to the mothers in general. It's a group health education not an individual one.

Interviewer: So, would you say that this is also one of the topics that is given for antenatal care?

Participant: I would say it is not a direct topic given but it comes in as a kind of secondary information probably when you are talking about how to live or have a healthy living within your house or home. It comes just as an example but it doesn't come as a major topic.

Interviewer: That is interesting, so why do you think it is not a major topic of health education especially when women come and them being informed that this can happen If you use this kind of firewood and this smoke can have this effect on you and how to avoid it when you think it is not a major topic for health education?

Participant: I think one of the reasons is that you know there are many topics which the midwives or the in charge can decide to bring forward and you find that they already have their man topics that they decide, you find a topic of HIV because HIV is a big issue pertaining testing, nutrition, family planning and birth preparedness and then danger signs. Those are the maIn things they talk about and because also probably you know people have a challenge before you give a topic you need to read and be well conversant with it. You don't just speak something out of the blues. So, people are already used to certain topics and some people fear going beyond outside those topics but it is very possible that it can become a major topic.

Interviewer: So how do we reach there?

Participant: How do we reach there? One, we need to ensure that the health workers who are the midwives and doctors they appreciate the topic of household air pollution. If they appreciate the burden of it then they can appreciate the worthiness or the value of that topic and I believe they can really intervene in it. Because you find things like nutrition it is because they have moved probably for certain trainings or there are some people who have come to check on and evaluate them and they keep on asking them nutrition, you get it and then they have to consider it that is

ok, they appreciate that nutrition is something which people need to learn. So, I believe that when we win organizing kind of, it can be a workshop with the people in antenatal from the various health facilities within and then share this with them, they can realize or appreciate and then I believe they can take up the initiative to add it into their major topics of health education.

Interviewer: Wawooh, this interesting that we make them try to know the burden so that they can appreciate the value of teaching about this. What else do you think would be needed for us to make health education about household air pollution routine topic for antenatal care health education talks?

Participant: One other thing I realized is that when we have to keep on making seasonal evaluations of how looking at the health education talks they have given because whenever you give a health education talk there is a book you have to document in. So, it can be a monthly evaluation of their health education, just going to look at the topics they have given and how they and in case how many times have they given a topic pertaining household air pollution and the target also needs to be the target population needs to be known because even in antenatal there are days allocated for people, you find there are days allocated for postnatal mothers, there are days allocated for mothers who are coming for the first time, there are various days allocated so we need to know the target population, which people do we really need to capture most and I believe if we need to have a big influence we need to start with those who are visiting for the first time there are higher chances that even probably somewhere during the course of antenatal care they can get to listen to more of the talks as reminders. So that s one thing I would suggest.

Interviewer: I think you have really gone into detail and I don’t know maybe if you have addition or if we are to design this program because it is a new thing, so if we are to develop it so that the people down there can take it up, what would be the required things?

Participant: You see sometimes mothers come late for antenatal visit; I mean they come on the right day but in the time of the day but in the time of the day, someone should come at like 8 but you find them coming at 10 to 11AM. Why? because they know they are going to give a health talk which is boring. Many times, health talks are given verbally without any charts. Usually you only find family planning having a chart and nutrition having a chart and those health talks of nutrition and family planning which have those charts really catch people’s attention because of the picture. There are many methods of learning: there is visual and then there are other ways. So, the point is we need to incorporate this into having teaching aids for household and the teaching aid could start with a story that can be read on the first page to provoke and draw their attention into the topic. Maybe a story of Mrs. so and so was pregnant and she was cooking in the house and from nowhere she had collapsed, the neighbor who came by knocked and no one was responding, kicked and found she had collapsed maybe she was cooking with charcoal or with wood or with even kerosene and they realized that ooooh there was maybe some air pollution and you can provoke what could have caused that death and you hear responses from people capturing them and then after that you bring up the topic with photos there showing

probably someone in the house cooking with maybe charcoal or kerosene and there is a lot of soot coming. Then they will start getting interested and trust me such a teaching method will capture them, will keep them hooked up for most of the session and they learn very well. For now, I believe we can use that, there is also an option of maybe using a video which probably could be expensive to shoot a video and then you have the video but then it is also not played by the midwife, it will need the player. So, it is also a very good method but its effectiveness has questions.

Interviewer: So, if right now we have developed a program and it is running, what do you think could be the challenges with implementing it?

Participant: Of course, I am not sure. I have not seen the project. One of things is we need to first of all, even as we develop this, we need to involve the health workers as much as possible which I appreciate this is already part of the involvement but also, we need to talk to the health workers since they are the ones who health educate. They are the ones who decide the topic like I said earlier on to me I believe a big turning point is when someone values or knows the burden of something, that is when they will appreciate and know it is serious. So, the way of communicating or sensitizing the health workers about this burden and to make them really appreciate it is one point we should consider. Because the participants, the mothers who will be coming for antenatal they have no choice other than to listen.

Interviewer: It is so interesting; it is the midwife who sets the guideline.

Participant: then the midwife should be well versed, should know the burden of it well and the take home message. So, if you have like that poster, it may be even big or like a booklet of it but a big one, a flip chart, it can be having even on the other side the take home messages/ the lessons to learn from this which reminds also the midwife who will be health educating.

Interviewer: The other issue would be the midwives who are going to be doing these health education talks, so what do you think their perception will be like if we are to talk to them and say that we want this to become a routine topic, do you think they will accept this topic to be part of the topics that they give in health education, the acceptability of this program?

Participant: The acceptability of it is ok but you know this is something which I realized on my own, ….(laughed…). So we need to, I don't know how we can do it whether there is some sort of motivation to simplify this but if not only the motivation can come there for the first few weeks when they have taken it up well, we phase it out gradually to make it sustainable that they will keep on teaching even without the motivation. But I think that is one of the ways we need to see, make them know that this is not much more work load we are giving them but it is an issue which we need to address and if possible and we can also have such papers presented in some conferences or in big conferences which have the ministry of health members in it then we can also suggest to them that this we need to adopt it into our health talks. You the ministry if you are to look at the essential newborn clinical care guidelines, they even have some sort of topics

you need to inform for health education, talk about family planning during this time, talk about in the first visit talk about birth preparedness. So, if we can approach them or use other people who sit there or even from within here in …I am aware there are people who sit on the decision-making table so these are things they need to appreciate but they only appreciate when it is evidence based and when the paper is already published there.

Interviewer: So, for acceptability they will be willing though some might consider it as extra work for them, how about the aspect of the message or topic its self. Do you think they find it appropriate or compatible with their beliefs and their values?

Participant: Yes, it is a little bit challenging because most of us have grown up in families where we go collect firewood or charcoal, by God's grace we have not done died but I am also aware there are other people who have died, even if we have not died. There is a way it can affect your health most especially when you are pregnant. You may not realize it, you have issues during pregnancy and blame it on something else but there are factors which can influence even in the long run it can affect you. I know some people will say but we grew up cooking even me I used to cook I was the one who used to cook a lot with firewood and nothing happened. It is like smoking you enjoy and nothing will happen for a short time but when you are pregnant something can happen you can lose the pregnancy early or have a preterm delivery or have hypertension come in or some other maybe non communicable disease come in but then you won't accept or link it up because it is not something which will happen all over. So, I believe that there are those people we have to be sure, there are midwives there are health workers or even doctors are there but the way we give them this information can help clear all those thoughts.

Interviewer: How do you think now those beliefs that we have used this and it is everywhere, how does it translate to their ability now to conduct the health education talk and inform people that this is not good or you can use this? How this translates to their ability to give the health talk?

Participant: When someone has not been informed prior about the magnitude of the problem, definitely it can affect their ability to give because someone, a mother may raise a question: healthcare worker you tell us to do this where should we cook from, how is it going to affect us, we have been cooking and there is nothing. So, how will such a health worker answer such a question when they have not appreciated the burden, when they have not known that the burden comes in with probably a long run but it can affect you in an indirect way you may not know. It is not like mosquitoes bite you and you get malaria after a week no, it may take a month. It may not happen during this pregnancy probably in the next pregnancy they need to appreciate that. So, if they have appreciated that I do believe that the uptake of this topic into health education would be very simple of which I believe that is the intention that you will ensure they appreciate it before starting to give the health talk.

Interviewer: So, are there any costs that are involved if we are to make this a routine topic?

Participant: The cost which will be involved will definitely be a onetime cost which is like I suggested if you are to adopt it will be printing/ making those posters and you will need someone creative to draw something realistic to have it. But also like I also suggested that it could be some motivation given to them may be every time whoever gives a health talk, the healthcare worker gets a bottle of soda at the end of the day or a bottle of water but with time you phase it out gradually.

Interviewer: So, in terms of barriers, the would-be barriers would money/finances.

Participant: The would-be barriers would be one finances has an implication but the knowledge I would say it, if I am to use the word sensitization or making the health workers aware of the burden would probably be another cost that is in terms of training.

Interviewer: I think here I was interested in knowing what could be the barriers if we are to make this program work, one could be the cost and another could be I don't?

Participant: The cost and the other barrier is now the negative attitude by the health worker which we can overcome.

Interviewer: The other issue would be that do you really think that the midwives would be able to regularly pass on this information during antenatal care health education talks?

Participant: Yes, I do think most especially when we have it in a target informant. I do think it is very possible when we have it in a target informant, they have appreciated it then probably there is a small motivation like a bottle of water for the beginning because the beginning is always not easy but it can help calm down their attitude.

Interviewer: So, you think it may not need to have something to push them like what, what is the opposite of the carrot?

Participant: No. because I also believe we need partnerships to implement this, we cannot just implement it as an individual, but can implement it. Even not as … alone, we need partnerships probably with some other people, with the hospital, with probably some other implementing partners.

Interviewer: So, I think we started by saying that this topic is not a major…, comes as a by the way or secondary, so what would you want to change in the future regarding how we give health education talks especially regarding this subject?

Participant: I would say this to be intentional; it needs to be an intentional topic by the health workers, they go sleeping knowing that tomorrow I have a topic on household air pollution to give, they wake up and prepare knowing that I have my poster around and I am going to give

this, yes, they are well aware of it, to me that is the intentionality which can have a great impact to the mothers.

Interviewer: So, I think I have now two questions you are remaining with, we discussed about the acceptability of midwives to give the health education talks, but about for the women, their acceptability to receive the information, what would you think about that?

Participant: The women look at the health workers as the people near God.when they tell you, you have to swallow this medicine two times in a day even when you do not know how the medicine is going to help to reduce the headache you will swallow it. So, if they listen and if the health worker brings it well, they will understand that yes, I know with probably the humble background I come from where we don't have a well-furnished kitchen. Modern kitchen, I am using firewood probably they will think of ways of using or maybe having a better kind of cooking kitchen somewhere but also, I know that they can also improve on their materials they use for cooking. So, do believe they will accept but I also do know there are some who will still be in doubt, in every population there are always people who will say that no what they are saying is not applicable. That is the big challenge by the way; the people in the community, certain things cannot be changed not because they do not want, someone may want to change but then the poverty within the community, the situation maybe she is a single mother, may be the husband is there but is a useless man and there is no way they can change the head and since they are not seeing a direct and immediate effect they may not change but they will appreciate, they will receive and know yes this is really important.

Interviewer: Wawooh, I think you have answered the next question of how they will take it and its compatibility with their beliefs and values, I don't know whether you have anything to add on that?

Interviewer: Ok, so the other thing is what opportunities do we have for then women who come for antenatal care in terms of their ability to adopt this message, now we are telling them maybe you can reduce exposure to smoke, maybe you can do this, which opportunities are there for them to adopt this message?

Participant: Opportunity to adopt think they are many, one of the things would be even them, first of the fast opportunity I would say there are health workers to teach, they are welcome, they are taught well and then the teaching aids they are there but then the other opportunity which we could also have implemented to be also having a small card like a small piece of paper just a summary and advise them on what they can do incase maybe they start experiencing some problems. Some of the effects of it, they should go to hospital and seek for care. I believe that if someone is given such a small piece of paper, not a very large piece, it is like if this is an A4 size, then a quarter of this to the women just some information on it maybe some pictures you know some people don't in Africa we don't like reading, so people like seeing photos so you

simplify for them, you can put there, just put maybe a phone number there maybe for further information you can call this number and you will be given more information.

Interviewer: The last thing is about motivations because now like firewood we use it almost all our life and now they are pregnant and now we are telling them this could affect them so if you want to avoid this effect on your pregnancy you can do this you can use may be a gas, you can use maybe this stove which does not produce much smoke or you can open the windows and what so that the smoke is not much, so what do you think is their motivation to adopt those messages especially being that they have been used to doing this all along?

Participant: I think one of the motivations for them to change could be then appreciating the trouble. It is like I will give you an example, when we have nets, some people say aaaaahhh the net makes me feel hot but once they appreciate that malaria kills and using a net can drastically reduce your rate of getting malaria, even if it is dry season and it is hot, someone will tough in their the net. So, the same belief or theory I will still employ here and tell them and say it is not a onetime data that you are going to give them and they accept and it is all done, it is a longterm thing, changing the mind is a process, it is continuous, so we should not give up in advocating for it but you cannot advocate for it alone, we also need partners to help us.

Interviewer: So, what do you think would be the impact of this counseling on pregnant mothers if we made it a routine topic?

Participant: I think the impact could be one or one of the impacts we will see is that some of the mothers will definitely stop using firewood or they will get other options or even if they can't get probably they will tell someone else be there and their exposure to it when cooking is reduced. That is one but of course the best would be kind of change and adopt other fuels which don't pollute and then also with time if there are certain conditions that come up with air pollution or certain instances we shall also see it drop even on the impact on the baby if the study is there to measure properly I think it would also show or be an indicator that well there is an impact happening in the community but also I would say the feedback itself from the mother would be something amazing.

Interviewer: Wawooh, this has been a very interesting discussion, I don't know whether you have any other issues you want to bring up regarding making this a routine topic otherwise we have come to the end of it.

Participant: Thank you so much, I have no any question.

Key informant interview with the healthcare worker

Interviewer: So, we can start, the first question is I wanted us to understand what is currently being done in antenatal care health education talks; do you think this is a routine topic that we give to our women about effects of smoke and cooking fuels?

Respondent: I don't know whether it is routinely given but I would say if you have already alerted them, I am sure they are giving that information to the mothers because smoking is one of the things that we were taught as midwives that a mother who is smoking, smoking can affect the growth of the baby. So, for us we have been thinking of the cigarette smoke, little did we know even this smoke can affect. But now that the study has shown, then that is really very big because it is practical, it is something that is very practical in our country and setting. People are cooking, they cook using firewood, charcoal and so forth, so you find smoke is available everywhere. So, I am sure this one is bringing the knowledge to even the midwives themselves, at least they know now that even this smoke can affect these children.

Interviewer: So, before we started doing this project, what do you think were some of the reasons why it was not a routine topic like the way we have anemia in pregnancy?

Respondent: I don't think it was a routine because first of all you know as … you many things to teach mothers. There are many things that are really causing risks to babies or their mothers, so you really want to capture. Sometimes you find may be the workload is too much and you find there is no time, but during history taking, now that you are taking history, you are able to tell the mother that by the way even this smoke is firewood and charcoal smoke, so it can have effect to the baby so you do this. So, I feel even that now is bringing knowledge to them.

Interviewer: So, the other question is if we are to make it routine topic like the way you do for anemia, danger signs, alcohol intake, what would this take us to make this topic about smoke and the effect it has on pregnant mothers?

Respondent: We could really integrate it in other health education talks and we make sure that it is in the program so that every time we are giving health education for example if we are giving it on a Monday when the new mothers are coming, you know first attendance is always on Mondays, so these are people, we should really give the information. Then the other ones who also come routinely you can give those ones as you are attending to them, but you know very well she attended the new clean (the first-time clinic). So, the re-attendance you know she has the idea, so that one becomes one on one. So, we could really integrate it into health education talks to mothers in first attendance.

Interviewer: Are there some other things that we might need for us to give that health education talks?

Respondent: Yes, we need the IC materials if we could make those IC materials to be available, we could show them what is happening: what do we mean by smoke, what is bringing smoke, how is it affecting the baby and mother. So, let us have those IC materials.

Interviewer: What of the midwives who are giving health education talks? What do you think they would need or what is required for them to be able to give the education talks?

Respondent: Ok if maybe you want to specify that you really want this one to be given, I really don't want to say that you have to pay them or what but it will be up to you if you feel that really it is necessary. But I would love you also to be coming to check on them to see that they are doing the right work, right thing and they are doing it regularly. You know there is saying that yes we are going to give and then you keep away but if could be coming some times to check on them and you see that it is routine. Because I know at one time even the ministry will take it up, I don't know whether you have already disseminated it. So, when we are starting with …, I think there will be time for us to disseminate this information and then it will become national.

Interviewer: Do you think midwives would find it acceptable to health educate mothers about effects of smoke on pregnancy?

Respondent: I think, the … I have are good … and I feel they will accept. I have seen how they have been doing before; I know they will accept to do it. The only problem is of course you know those other challenges like workload and so on and so forth. Those are the only things but if they could use even the students who come, talking about smoke is not a problem, any person can do even the student if they can only teach the student how to do it I think we shall benefit from it.

Interviewer: I think that will sort out the issue of workload, so they can maybe do other tasks as students are also doing.

Respondent: And then maybe we do some health education talks on the radios if we could also in future put them there so that you also talk about effect of smoke to babies and mothers. I think that one will come at a later time but it will be another way of educating communities.

Interviewer: So, if we are to make it part of our antenatal care education sections, are there any costs that will be involved?

Respondent: Health education talks do not always have costs apart from may be developing the IC materials, that one can be costly I know it will need you to ……………Then it depends on you now who is doing your research, if you are happy you can always tell them I have some tea here for you like that. You know when somebody is motivated; you find somebody is struggling to do his/her best. This person is motivating us let us do it better, maybe just that because otherwise something which is benefiting a mother and a baby, I don't think anybody would really sit on it.

Interviewer: So, apart from costs because I was thinking of cost implications, are there any barriers that we might face if we are to make this a routine topic?

Respondent: Now apart from these are other costs, even staffing is becoming a problem. For example, if the students are not there, another staff has gone maybe for a workshop, this one has gone there, but if the person who is there can assign a day to give this health education talk it would be better so that it becomes a routine. So, at least is given every week, if we give it once a week, it’s okay but we need to capture which mothers are we giving, is it the new mothers or re attendance but for me I will propose the new mothers.

Interviewer: Then the other issue you mentioned was regular education, so would you think the midwives would be able to provide it regularly or the intervals that you have talked about?

Respondent: They can, as I said a… takes the history of a mother, when you are taking the history of mother like are you smoking? really is that a question we can ask African Ugandan mothers. Really? Here in Uganda no because smoking is not common with our mothers. We don’t. In fact, there are no women here smoking, I think maybe there in the city but here it is strange. So, the smoke we shall be asking is about the firewood. It is interesting, so it is about the smoke of the firewood and charcoal, that is what we are going to do. Meaning even you the researcher in future we can include that because not only cigarette smoking, we include in antenatal other varieties of smoke.

Interviewer: So instead of asking about cigarette smoking which is not common, we can ask about the use of firewood, charcoal and so on (anything that generates smoke). So, when you have identified one when you are taking history and that question, I think is there in that HMIS.

Respondent: It is there in antenatal card, so we would modify it and put that also.

Interviewer: So, we are almost coming to the end, then the other thing is about what you would like to see in the future or what you would want to see change in the future regarding informing mothers about the effects of cooking fuels on their pregnancy?

Respondent: So, I know this charcoal and firewood is part of us, that is what we use for cooking, in future I want to see at least if there has been a problem to these babies, I want to see that there is a change. I want to see that there is reduction in premature delivery and so forth. I want to see that because underweight also affects children and they are born small, so I want to see that it has reduced and then at least people are informed such that when you ask them, they know that it is not the cigarette alone even this other smoke can cause problems. Then of course I want to see maybe in future the effect of that smoke to the mother herself, does it also cause any problem to fertility or….., we need to explore and find out, apart from causing problems to the baby, is there anything it causes to me apart from infection to the lungs, maybe hypoxia, you start getting dizziness faaaaaah (just like that), you even don’t know why maybe it is the smoke. So that is what I want.

Interviewer: Then the second last question is about pregnant women and their families, when they come for antenatal care, do you think they will be willing to receive this kind of information about cooking fuels and the impact it can have or how to avoid the smoke?

Respondent: Yes, those who escort mothers, the women, husbands, maybe their mothers in law, they may be willing but of course be ready to answer many questions because they will tell you what do we do now because those are the things we use, but of course we are not telling them to stop suing them. We are telling them while you are using this let it be dry firewood that is making good fire, not this one that is making smoke and if it is making smoke, open the windows, open the doors, cook early so that you are not locked in the house to cook at night, cook early at day time and you see that your baby is growing well and you are also healthy. So, I think they will be willing, we may need only to summarize our talk in such a way that it is short, you don't take it very long, make it short, meaningful and you see that they are interested.

Interviewer: So, you said that sometimes you may be reluctant

Respondent: When you talk all the stories, they will start saying now ok, but when you bring the points and then in future after some films, show some of these things how it affects because I know there are some videos somewhere, show them this is what happens.

Interviewer: So, the last question is about what might affect them if they are to adopt the messages that we have given them? What do you think are some of the problems that they might say that now … you have told us about this but now we can't do it?

Respondent: Now it depends how they are going to perceive it. Others of course are going to take it positively, the ones who are taking negatively are the ones who are going to have problems because first of all they will misunderstand you. Others may think you are saying stop, aaah they are saying it is not good then they go and condition their husbands that they are saying don't use this it is going to…… So it depends now on the person who is giving the health education talk, it depends on our IC materials that we give. We need to have some of these brochures. We can develop them, the ones who can read you give them and tell them read, knowledge is power just encourage them. Those are the things now; it might affect them positively or negatively.

Interviewer: How about some enablers or facilitators for them to adopt the messages that we give them like we are telling them to use firewood which does not produce too much smoke or use a gas or opening windows, are there some enablers that can enable them to adopt those messages?

Respondent: I may not be so sure whether they are there but it depends now on an individual mother. Are you able to pay this or buy this? Let us make it in such a way that it does not demand so much, it shouldn't over demand that you must use gas, you must use what. No. let us make it in such a way that first of all it brings knowledge to them, they know that wet firewood is bad and if it is making smoke, dodge it don't use it, you a mother should either be aside or

open the windows but don’t be at the smoke, it affects your lungs and you baby, it affects the growth of the baby even the brain.

Interviewer: Thank you ….for the interview, I don’t know whether you have any other things you might want to bring out regarding what we have discussed.

Respondent: I don’t have anything. I want to see that really this study should make a change to these mothers. Let it not stop just there as study, let it be something which is going to be a change agent.
